# Effectiveness of Pharmacokinetic-Guided Hydroxyurea Dose Individualization in Patients with Sickle Cell Anemia: A Mini-Review

**DOI:** 10.3390/ph16060857

**Published:** 2023-06-08

**Authors:** Joelma Santana dos Santos Neres, Sètondji Cocou Modeste Alexandre Yahouédéhou, Marilda Souza Goncalves

**Affiliations:** 1 Laboratório de Investigação em Genética e Hematologia Translacional, Instituto Gonçalo Moniz, Fiocruz-BA, Salvador 40296-710, Brazil; 2 Laboratório de Pesquisas em Anemia, Faculdade de Farmácia, Universidade Federal da Bahia, Salvador 40170-110, Brazil

**Keywords:** sickle cell anemia, hydroxyurea, personalized dose, pharmacokinetics profile

## Abstract

Inconsistent therapeutic responses have been observed among patients with sickle cell anemia (SCA) undergoing hydroxyurea (HU) following the adoption of the standardized protocol. Moreover, this treatment regimen necessitates a prolonged period to reach the maximum tolerated dose in which beneficial therapeutic effects are observed in most SCA patients. To overcome this limitation, several studies have performed HU dose adjustments in SCA patients based on individualized pharmacokinetic profiles. The present systematic mini-review aims to select and analyze published data to present an overview of HU pharmacokinetics studies performed in SCA patients, as well as evaluate the effectiveness of the dose adjustment strategy. A systematic search was performed in the Embase, Pubmed, Scopus, Web of Science, Scielo, Google Scholar, and the Virtual Library of Health databases from December 2020 to August 2022, with a total of five studies included. Inclusion criteria consisted of studies in which the dose adjustment was performed in SCA patients based on pharmacokinetic parameters. Quality analyzes were performed using QAT, while data synthesis was performed according to the Cochrane Manual of Systematic Reviews of Interventions. Analysis of the selected studies revealed improved HU treatment effectiveness using personalized dosages in SCA patients. Moreover, several laboratory parameters were utilized as biomarkers of the HU response, and methods designed to simplify the adoption of this practice were presented. Despite the scarcity of studies on this topic, HU-personalized treatment based on individualized pharmacokinetic profiles represents a viable alternative for SCA patients who are candidates for HU therapy, especially for pediatric patients. Registration number: PROSPERO CRD42022344512.

## 1. Introduction

Sickle cell anemia (SCA) is an autosomal recessive genetic disease characterized by the inheritance of the hemoglobin S variant (HbS) from both parents. HbS results from the substitution of adenine for thymine in the sixth codon of the beta (β)-globin gene (*HBB*), leading to the replacement of glutamic acid with valine [1]. Due to the polymerization of HbS under recurring conditions of hypoxia, red blood cells thereby assume a sickle shape [2], and suffer two main events: (1) hemolysis, characterized by the rupture of red blood cells with a release of hemoglobin, reduction in the concentration of nitric oxide (NO), and increase in the inflammatory process and activation of endothelial cells [3], and (2) the vaso-occlusion, in which sickled red blood cells adhere to endothelial cells along with reticulocytes, platelets, and leukocytes, leading to obstruction of blood vessels [1]. These events are responsible for several clinical manifestations as acute chest syndrome, priapism, pain crises, malleolar ulcers, stroke, and pulmonary hypertension [2].

Hydroxyurea (HU), commercially known as HYDREA^®^, is one of the drugs approved by the U. S. Food and Drug Administration to treat SCA patients with more severe clinical profiles [4]. Despite the broad indication and usage, the mechanism of action associated with HU has yet to be completely elucidated. Reports showed that HU inhibits ribonucleotide reductase, maintaining cells in the S phase of the cell cycle and preventing the continuation of cell division. Moreover, it activates soluble guanylate cyclase through the production of nitric oxide (NO), leading to an increase in the production of cyclic guanylate monophosphate, reactivation, and transcription of the gamma (γ)-globin gene (*HBG*), and, consequently, the production of fetal hemoglobin (HbF) [5]. Another point that needs to be fully elucidated is its pharmacokinetics. Reports have shown that HU is hydrophilic and has a volume of distribution close to that of water, with approximately 37% of the orally administered dose being excreted through urine without being metabolized [6]. Some authors defend the partial excretion of the drug by the kidneys and metabolism by the liver via enzymatic reduction catalyzed by the cytochrome P450 monooxygenases (CYPs) [4], which transform HU into urea and carbon dioxide [7], in addition to a portion which is converted into NO in both the liver and blood [8]. However, other studies have argued that HU is mostly excreted by non-renal mechanisms [9,10].

Currently, the standard therapeutic strategy is to start treatment with an initial fixed dose of 15 mg/kg/day, with increases of approximately 5 mg/kg/day every 4 to 8 weeks until reaching a dosage of 30 to 35 mg/kg/day, determined as the maximum tolerated dose (MTD) identifying the absolute neutrophil count of 1.5 to 3.0 × 10^9^/L, where evidence of the therapeutic effectiveness of HU has been noted, such as increases in the hemoglobin concentration and mean corpuscular volume (MCV), as well as the HbF level [11]. Despite the known beneficial effects of HU therapy in SCA patients, the need for dosage adjustments demands a higher number of medical visits, and, therefore, an elevated frequency of laboratory parameter monitoring. Furthermore, the variability in the therapeutic responses represents a significant challenge and may arise due to several factors, including variability in the pharmacokinetic profile of the patients.

Accordingly, several studies have sought to investigate the pharmacokinetic profile of HU in SCA patients, and based on an established profile, administer a personalized HU treatment regime for reducing variability in the therapeutic response [6]. The present review aims to select and analyze these studies to present an overview of HU pharmacokinetics in SCA patients, as well as analyze the effectiveness of personalized dosing.

## 2. Results and Discussion

### 2.1. Selection and Qualitative Analysis of Articles

**Figure 1:**
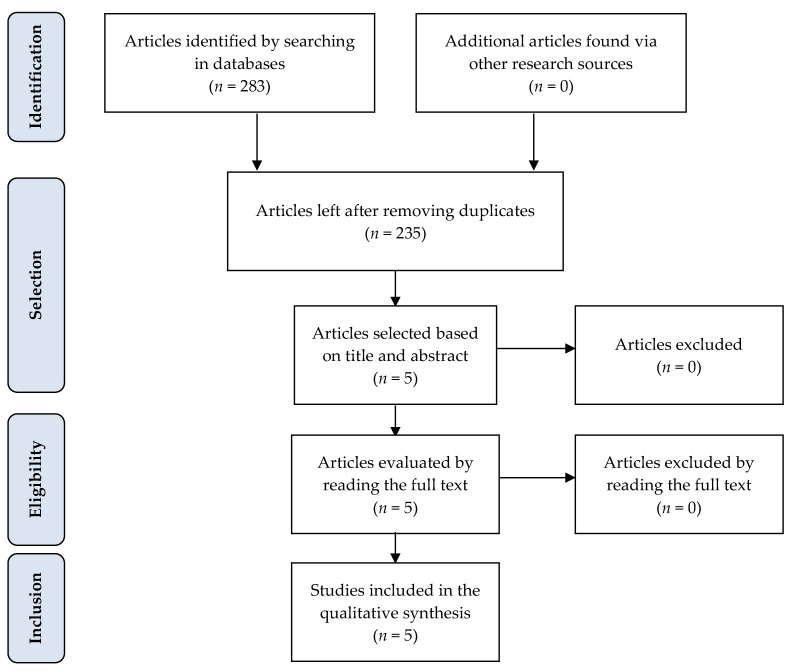
Flowchart detailing the present study protocol in accordance with the PRISMA model.

Articles were selected following the protocol illustrated in the flowchart by the Preferred Reporting Items for Systematic Reviews and Meta-Analyzes (PRISMA) study (Figure 1). Initially, 283 articles corresponding to the selected search terms were identified in different databases, of which 48 were duplicates. Two hundred and thirty articles were excluded based on title and abstract screening, as these were unrelated to the topic of the present review (Supplementary Material). Finally, the selected articles (*n* = 5) were fully analyzed and included in the qualitative synthesis, namely: Dong et al., 2015; McGann et al., 2019; Meier et al., 2020; Paule et al., 2011; and Quinn et al., 2021.

Using the Quality Assessment Tool for Quantitative Studies (QAT) [12], we obtained the results of the qualitative analysis performed, analyzing aspects related to the methodology of the studies: selection bias, study design, and the data collection method. Dong et al., McGann et al., Quinn et al., and Meier et al. articles were classified as strong. The study of Paule et al. was classified as moderate since fewer than 60% of the patients completed the study.

### 2.2. Main Findings from the Selected Articles

All included studies performed HU dose adjustments based on pharmacokinetic parameters in SCA patients and demonstrated treatment effectiveness using laboratory parameters. Table 1 presents the objectives and main results of each study.

Dong et al. conducted a study involving 96 SCA patients treated with 20 mg/kg of HU. The median age of the patients was 9 years of age [13]. These authors investigated individual pharmacokinetic profiles using a non-compartmental pharmacokinetic model. Data revealed an association between the AUC_0-∞_ of 115 mg L^−1^ h^−1^ and the MTD of HU, suggesting that this value could be achieved using the MTD. In addition to the HU population pharmacokinetic model, an optimal sampling strategy for determining an individual’s pharmacokinetic profile using only three sampling times was developed, with serum cystatin C and body weight identified as significant predictors of HU clearance.

The study by McGann et al. was conducted in children with SCA (*n* = 51) (age range: 6 months to 21 years of age) who were treated with 20 mg/kg of HU [14]. A pharmacokinetic-guided dose individualization strategy based on the population pharmacokinetic model permitted MTD achievements more efficiently, and a Bayesian adaptive control strategy was employed to measure the systemic exposure to HU via a spaced sampling schedule. Data demonstrated the influences of age and renal hyperfiltration on the pharmacokinetic profile of HU. Moreover, the time to reach MTD was found to be shorter, and dose determination, in accordance with pharmacokinetics produced better results, which was assessed via biomarkers including HbF and hemoglobin.

Recently, Quinn et al., published a study that is the continuation of that performed by McGann et al. They selected 48 children from the 51 included in the previous study who had F-cell analysis performed at least once. Early initiation of HU at the predicted MTD using pharmacokinetics-guided starting doses yielded promising results. Children showed sustained and near-pancellular or pancellular HbF distribution, indicating the effectiveness of this therapeutic strategy in meeting or exceeding the goals intended for SCA “curative treatment” [15].

Similar to the studies cited above, Meier et al., are planning to include a minimum of 104 pediatric SCA patients, with the age ranging between 6 months and 21 years of age [16]. These children will be divided into two groups: one will receive an initial dose of 20 mg/kg/day, while the second will receive an initial dosage guided by pharmacokinetics. In their study, the amount of blood to be collected will be optimized using a volumetric absorptive microsampling (VAMS) device to enhance the therapeutic response.

Another study performed by Paule et al. considered SCA patient data from two previous clinical studies. The first was a PK-PD study that analyzed blood samples from 81 adults aged 18 to 54 years old who received an initial HU dose of 20 mg/kg/day. The second was a study that analyzed the bioequivalence of a new formulation of coated tablets in 16 adult SCA patients [17]. Based on a population pharmacokinetic model, the authors observed variability in the pharmacokinetic parameters, such as the volume in the central compartment, clearance, absorption constant, and distribution constant.

### 2.3. Dose Adjustment Using the Population Pharmacokinetic Model

Considering the search terms chosen, the intervention employed in all selected articles was HU dose adjustment based on pharmacokinetic parameters. This conduct was prompted by the ineffectiveness of conventional HU treatment as was previously observed in some patients who received HU at an initially fixed dose followed by escalation to MTD, which may be due to inter-individual pharmacokinetic variations [18,19]. Moreover, other disadvantages, such as recurrent laboratory testing and prolonged time to reach MTD, further contribute to the non-adherence to pharmacotherapy, together with family members’ fear of cancer development as well as other adverse effects [18].

Pharmacokinetic analyzes in the selected studies were performed using the population pharmacokinetic model, which requires fewer samples per individual and enables the analysis of the studies which generate dispersed and uneven data, e.g., those performed in pediatric populations [20]. Moreover, Dong et al. implemented non-compartmental pharmacokinetics in their study.

### 2.4. Sample Collection for Pharmacokinetic Analysis

For their study, Dong et al. performed plasma sample collection at seven different times, ranging from before HU therapy to 8 h after administration [13]. Moreover, these authors also developed an experimental design, which identified more informative collection times, thereby reducing the number of samples that needed to be collected for future HU pharmacokinetic studies: 15–20 min, 50–60 min, and 3 h after HU administration, respectively. These identified times help to determine the HU dose by pharmacokinetics as they reduce the number of collections, which is a relevant challenge, especially in pediatric patients. Due to this determination and the establishment of a standard AUC for predicting MTD, this study has been used as a basis in the design of subsequent studies performed by other authors including Estepp et al. [21], McGann et al. [14], and consequently Quinn et al. [15], and Meier et al. [16].

In addition to the collection periods determined by Dong et al., Meier et al., used VAMS devices following the development of a method based on tandem mass spectrometry. This required smaller sample volumes and may serve to facilitate collection in pediatric patients as well as dose adjustment by pharmacokinetics.

In the case of the study conducted by Paule et al., samples analyzed from the first study were collected on the first day of HU treatment, and then at fifteen days, followed by after the first, second, fourth, and sixth subsequent months. In the second study, samples were collected before HU administration and 45, 90, 120, 150, 180, 240, 360, and 480 min later [17]. It is worth mentioning that among the included articles, this was the only study that was performed in adult patients, which may have resulted in a more straightforward collection. Analysis of laboratory parameters showed that patients treated with an HU-personalized dose presented significantly increased HbF levels compared to patients who followed a traditional dosage regimen.

### 2.5. Pharmacokinetics and Pharmacodynamic Parameters

Several important pharmacokinetics parameters encountered in the included studies are presented in Table 2. Dong et al. demonstrated a mean AUC_0-∞_ value of 115.7 ± 34.0 mg L^−1^ h^−1^ at MTD, establishing 115 mg L^−1^ h^−1^ as the predicted AUC_0-∞_ at MTD. They also reported MTD values ranging between 14.2 and 35.5 mg/kg/day [13]. This finding corroborates the McGann et al. study, which identified a narrower MTD range (26.7 ± 4.8 mg/kg/day) [14]. On the other hand, Paule et al. presented dosages ranging between 500 and 2000 mg, with 78% of the patients receiving 1000 mg [17]. Another aspect that is also of utmost importance to be considered is that the MTD in patients undergoing dose adjustment according to the standard protocol was reached after 8 months of dose escalation [21], while the patients in the McGann study achieved MTD in just 4.8 months [14]. Although, in the later study, the variation of the time was large (from 3.3 to 9.3 months), the findings demonstrated the effectiveness of the pharmacokinetic dose adjustment regimen in shortening the time necessary for patients to reach the ideal dosage (MTD) when compared to the traditional dose adjustments (4.8 months vs. 08 months, respectively). Moreover, these studies demonstrated clearance values that were remarkably similar except for Dong et al., which observed a slightly higher value than the others. However, the authors considered this value to be close to that described in the literature for both adults and children [13].

Regarding pharmacodynamics, the studies included in the present review identified some covariates, such as body weight, which was associated with clearance and apparent volume of distribution [13,14]. This finding was also identified in a previous study [22] and demonstrates the importance of body weight in assessing the pharmacokinetics of HU in individuals. Dong et al., also reported cystatin C but not serum creatinine as a covariate, as they observed its association with the glomerular filtration rate, kidney injury, and impaired HU excretion, and was thereby considered as a crucial predictor of HU clearance [13]. Moreover, cystatin C has been considered as an important biomarker of renal function in patients with SCA, as pathology can lead to glomerular hypertrophy, and levels of cystatin C have been found to correlate with the glomerular filtration rate [23].

Another covariate considered as predictor of the HU response was HbF, in addition to other laboratory parameters such as hemoglobin and reticulocyte count, which were found to be more pronounced in younger patients who received individualized dosage [14]. Likewise, Meier et al. observed an increase in HbF levels in patients who underwent dose adjustment via pharmacokinetics compared to those who received an initial dose of 20 mg/kg/day [16]. Moreover, these authors also considered pharmacodynamic parameters, such as hemoglobin, MCV, and total reticulocyte and neutrophil counts.

The study of Paule et al. revealed increases in HbF levels and MCV, and reductions in bilirubin, LDH, and neutrophil count after six months of treatment [17]. These authors also reported that HbF achieved a steady state long after MCV, with higher inter-individual variability and responses observed in the simulation of a dosing regimen in which HU was administered/day compared to a five-day per week consecutive regimen.

In addition to the laboratory biomarkers, Quinn et al. demonstrated that near-pancellular and pancellular HbF expression can be used as a biomarker of HU therapy effectiveness. They established the total F-cell fraction of 80% and the F/F-cell values of 10 pg as optimal values corresponding to better pharmacological or genetic responses.

As shown in Table 2, the Paule et al. study is the only one which involved adults. This is an important variable that needs to be considered, since there are existing differences in the pharmacokinetic processes between children and adults, such as the decrease in the intestinal transit time that affects absorption, and the increase in the distribution of hydrophilic drugs as HU, due to the greater volume of extracellular water in preschool children [20]. Moreover, drug metabolism can be influenced by age and liver clearance, which is higher in children than in adults due to elevated liver blood flow. The expression level of liver enzymes such as CYPs, which were found to be positively correlated with age, is another factor that may affect drug metabolism [19]. Despite this, we observed similarities in the pharmacokinetic and pharmacodynamic parameters in the presented studies.

### 2.6. Limitations and Perspectives

The main limitation of the present study is the number of included studies, based on the search terms we established to meet our objective. Moreover, none of these studies stratified patients according to genetic factors such as β^S^-globin gene haplotypes, which are associated with variations in HbF levels, given that this parameter is used when assessing the HU response. Another limiting factor is the relatively fewer participant number in some of these studies. However, this may be due to the complexity of pharmacokinetic studies themselves, principally those involving children, in addition to other issues related to therapy adherence, withdrawal during the study, etc.

**Figure 2:**
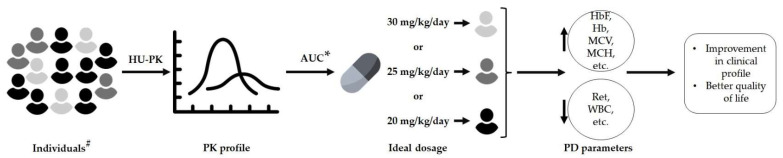
HU-personalized treatment based on individualized pharmacokinetic profiles and its effectiveness in SCA patients. HU-PK: hydroxyurea pharmacokinetics, AUC: area under the curve, PD: pharmacodynamic, Hb: hemoglobin, HbF: fetal hemoglobin, MCV: mean corpuscular volume, MCH: mean corpuscular hemoglobin, Ret: reticulocyte, WBC: white blood cell, #: individual candidates for HU therapy, with different genetic backgrounds, from distinct geographical regions, and with different ages, and *: determination of the ideal dosage using the AUC expected at the maximum tolerated dose (115 mg L^−1^ h^−1^).

According to the above, there is a need for studies involving different populations, considering aspects such as the genetic background of geographical regions, age groups, etc. These studies will shortly enable, the application of this therapeutic strategy in patient candidates for HU therapy, bringing them substantial benefits. Figure 2 clearly illustrates this perspective.

## 3. Methods

The present systematic mini-review investigated previously published studies based on the following question: “Does individualized dosing in accordance with pharmacokinetic analysis contribute to the effectiveness of HU treatment in SCA patients?”.

Inclusion criteria were clinical trial-type articles performed in SCA patients where the intervention was HU dose adjustment, regardless of HU formulation and participant age, and without limitations as to the publication language or date. Searches between December 2020 and August 2022 on the Embase, Pubmed, Scopus, Web of Science, Scielo, Google Scholar, and the Virtual Library of Health databases were performed using the following terms: (“sickle cell disease” OR “sickle cell anemia” OR “sickle cell anaemia”) AND (“hydroxyurea” OR “hydroxycarbamide”) AND pharmacokinetics AND (“dosing regimen” OR “guided dose” OR “guided dosing”) and their equivalents in Portuguese, Spanish, and French. Characteristics of each study were listed and compared with the inclusion criteria to assess their eligibility.

Data selection and study quality analysis were performed by two independent authors using the QAT [12] that evaluates the aspects as risk of bias and study design, while data synthesis was performed according to the Cochrane Manual of Systematic Reviews of Interventions. We extracted the data from the included studies and summarized them in a table (Table 1) including variables such as country, objectives, participant’s characteristics, pharmacokinetic model, and principal results. We also sought to investigate and report pharmacokinetic parameters such as the AUC and MTD, and pharmacodynamic parameters including HbF, MCV, and hemoglobin. The protocol has been registered in PROSPERO CRD42022344512.

## 4. Conclusions

Analysis of the included studies highlights the effectiveness of dose adjustment based on pharmacokinetic parameters, which may contribute to greater patient adherence in addition to improvements in the HU response, as evidenced by the pharmacodynamic parameters, and the reduction of clinical manifestations in comparison with conventional HU treatment. Several crucial studies presented strategies that resulted in less blood volume and fewer collection events to enable the adoption of this practice. They also identified an AUC value as an MTD predictor. However, additional studies are required, since only a few studies were identified as consistent with the objectives of this review.

## Figures and Tables

**Table 1:** Objective and main results of the included articles.

Study	Country	Objective	Number of Participants	Principal Results
[13]	USA	Develop an HU pharmacokinetic model in a pediatric population in combination with Bayesian estimation to individualize HU dosages in pediatric patients with SCA.	96 children	Development of a guided dose strategy, which requires three plasma samples collected at 15 to 20 min, 50 to 60 min, and 3 h after the initial dose, respectively, with exposure being estimated using Bayes’ theorem incorporating previous pharmacokinetics data; Prediction of a area under the curve (AUC_0-∞_) value (115 mg L^−1^ h^−1^) corresponding to MTD; Significant changes in the maximum velocity and apparent volume of distribution according to body weight; Significant association in elimination parameters, such as cystatin C and the glomerular filtration rate.
[14]	USA	Simplify and decrease HU dose-escalation so that children receive an optimal dose based on individualized pharmacokinetic parameters.	51 children	Variability in HU absorption, noting the influence of age on pharmacokinetic processes secondary to renal hyperfiltration; Reduction in time to reach MTD compared to other studies; Improvement in all hematological parameters after 6 months of treatment; Demonstration of a robust and sustained HU response in patients with individualized dosages guided by pharmacokinetics when started early, during the first years of life, in addition to shortening the clinical visitation time to 3 h.
[15]	USA	Investigate the utility of flow cytometric F-cell analysis and demonstrate sustained and near-pancellular or pancellular HbF distribution in SCA children undergoing an early and individualized pharmacokinetics-guided dosing of HU.	48 children	SCA children had sustained near-pancellular and pancellular HbF expression, which did not depend on the genetic determinants of HbF expression; Early HU use in children before 3 years old and better medication adherence increases the chance of sustained and pancellular HbF expression; The total F-cell fraction of 80% and the F/F-cell values of 10 pg have been identified as optimal values corresponding to better pharmacological or genetic responses.
[16]	USA	Validate the feasibility and benefits of the pharmacokinetic-guided dose approach in a multicentric study.	104 children	Study is currently in progress, and therefore no results have been presented; The study hypothesis is that pharmacokinetic-guided dosing will improve laboratory responses compared to weight-based dosing, as the study outcome will be relevant for optimizing treatment in children with SCA.
[17]	France	Develop PK-PD population models for HU, seeking to assess the exposure-efficacy relationship and variability. Compare continuous and interrupted dosing regimens and develop recommendations for monitoring treatment response.	97 adults	Significant inter-individual variability was found in several parameters, such as volume in the central compartment, clearance, absorption constant, and transfer constant from the central to the peripheral compartments; Increases in HbF and MCV, as well as decreases in bilirubin and LDH during treatment with HU; Variation in the intensity of the effect provided by HU in patients. Those undergoing a continuous-dosing regimen presented small and greater increases in MCV and HbF, respectively, compared to patients treated using an interrupted-dosing regimen.

SCA: sickle cell anemia, HU: hydroxyurea, AUC_0-∞_: area under the curve from time 0 to infinity, MTD: maximum tolerated dose, PK: pharmacokinetics, PD: pharmacodynamics, HbF: fetal hemoglobin, MCV: mean corpuscular volume, LDH: lactate dehydrogenase, and USA: United States of America.

**Table 2:** Pharmacokinetics and pharmacodynamic parameters of the included articles.

Study	Age (Years)	Gender	Health	Biomarkers	AUC_0-∞_	CL	MTD	HbF	MCV
		(Male/Female)	Conditions		(mg L^−1^ h^−1^)	(Lh^–1^ 70 kg^–1^)	(mg/kg/Day)	(%)	(fL)
[13]	1.9–16.5	42/21	SCA	Serum creatinine, direct glomerular filtration rate, serum cystatin C, urine albumin, HbF	91.1–115.7	19.56	14.2–35.5	--	--
[14]	0.5–21.0	29/21 *	SCA	HbF, Hb, MCV, ARC, WBC, ANC, platelets	67–91	9.7–14.0	26.7 ± 4.8	33.3 ± 9.1	91.9 ± 15.3
[15]	2.3–21.9	27/21	SCA	HbF, Hb, Hct, MCV, ARC, RDW, ANC, platelets, F-cell, F-reticulocytes, F/F-cell	67–91 **	9.7–14.0 **	17.8–38.6	≥31.5	≈ 70–120 ^¥^
[16]	xx	xx	SCA	Reticulocyte count, HbF, cystatin C	xx	xx	xx	xx	xx
[17]	18–54	29/68	SCA or S/β-thalassemia	Hb, HbF, MCV, MCH, PMN, platelets, bilirubin, LDH, ferritin, AST, ALT, creatinine, urea	--	10.4–12.9	--	3.9–41.6	81–131

AUC_0-∞_: area under the curve from time 0 to infinity, CL: clearance, MTD: maximum tolerated dose, Hb: hemoglobin, HbF: fetal hemoglobin, Hct: Hematocrit, MCV: mean corpuscular volume, MCH: mean corpuscular hemoglobin, ARC: augmented renal clearance, RDW: Red Cell Distribution Width, WBC: white blood cell, ANC: absolute neutrophil count, PMN: polymorphonuclear leukocytes, LDH: lactate dehydrogenase, AST: aspartate aminotransferase, ALT: alanine aminotransferase, --, no information available, and xx, no information because the study is undergoing. * One family decided to not initiate HU after enrollment of their child, so the analysis included only 50 children who started HU. ** Data is from McGann et al., 2019. ^¥^ Data was collected from the figure provided in the article.

## Data Availability

Not applicable.

## Supplementary Materials

The following supporting information can be downloaded at: https://www.mdpi.com/article/10.3390/ph16060857/s1, Table S1: List of the excluded articles.
